# Rapid identification of *Sporothrix brasiliensis* by MALDI-TOF MS directly from clinical cultures in an Amazonian epidemic setting

**DOI:** 10.1007/s42770-026-01996-8

**Published:** 2026-07-01

**Authors:** Daniel dos Santos Caldas, Gabriel Silas Marinho de Sousa, Pedro Henrique Oliveira Favacho, Rodrigo Santos de Oliveira, Elaine Patrícia Tavares do Espírito Santo, Silvia Helena Marques da Silva

**Affiliations:** 1 Programa de Pós-Graduação em Biologia de Agentes Infecciosos e Parasitários, Instituto de Ciências Biológicas, Universidade Federal do Pará, Belém, 66075-110 Pará Brazil; 2 Laboratório de Micologia Médica, Seção de Bacteriologia e Micologia, Instituto Evandro Chagas - IEC/SVSA/MS, Ananindeua, 67030-000 Pará Brazil

**Keywords:** Sporotrichosis, Filamentous phase, Rapid diagnosis

## Abstract

Sporotrichosis caused by *Sporothrix brasiliensis* is a highly virulent zoonotic mycosis rapidly expanding in Brazil, with increasing records in Pará since 2018. The reference diagnosis relies on culture and the time-consuming induction of fungal dimorphism, which delays taxonomic confirmation for weeks. The objective of the study was to develop and validate an in-house spectral library by MALDI-TOF MS for the rapid identification of *S. brasiliensis* in the Amazon region of Pará, prioritizing the feasibility of direct identification from the filamentous phase (primary growth). Thirty-seven Main Spectral Profiles (MSPs) from regional isolates were created, with 24 derived from the yeast phase and 13 from the filamentous phase. For validation, 46 molecularly characterized isolates were used in both filamentous and yeast-like morphologies, followed by the application of 39 routine clinical isolates obtained from primary culture. The library achieved 100% accuracy (score ≥ 2.0) in species identification for both fungal phases. Notably, all 39 routine clinical isolates were correctly identified directly from the filamentous growth, reducing the diagnostic time to an interval of 5 to 7 days. Although the yeast phase presented superior scores and greater proteomic stability, the mycelial signature proved robust for clinical use. The customized library overcomes the gaps in commercial databases and establishes a high-throughput workflow for the surveillance of *S. brasiliensis*. This technical optimization allows for more agile One Health surveillance, which is essential for epidemiological mapping and the control of zoonotic expansion in the region.

**Supplementary Information:**

The online version contains supplementary material available at 10.1007/s42770-026-01996-8.

## Introduction

 Sporotrichosis is caused by pathogenic species of the genus *Sporothrix*. In recent years, this disease has become a subcutaneous mycosis of global importance and relevant to public health [1, 2]. In Brazil, the scenario changed drastically after the emergence and rapid expansion of *Sporothrix brasiliensis*. This species is highly virulent and associated with zoonotic transmission, especially among domestic felines [3–5]. Unlike classic sapronoses associated with *S. schenckii* and occupational activities, infection by *S. brasiliensis* has the potential for urban outbreaks and severe cases (epizootics and epidemics) [3, 6, 7].

The current epidemiological scenario highlights the internalization and expansion of the pathogen to new areas, including the Brazilian Amazon region [8]. Reflecting this growing public health threat, sporotrichosis has recently been declared a compulsorily notifiable disease in Brazil. In Pará, a growing increase in the number of human and animal cases is observed, with the first official records in the municipality of Belém dating back to 2018 [9, 10]. In these regions, access barriers to diagnosis in peripheral areas underestimate the actual incidence of the disease. In the vast Amazon, it becomes essential to adopt precise and accessible identification technologies to strengthen local surveillance [8, 9].

The laboratory diagnosis of sporotrichosis still faces major bottlenecks. The reference method remains isolation in culture, combined with morphological characterization [11, 12]. This process is time-consuming, normally requiring induction of fungal dimorphism for confirmation, which can take weeks [6, 13]. On the other hand, molecular methods (species-specific PCR and sequencing), although accurate, are expensive, technically complex, and time-consuming, especially in assays with a large number of clinical samples [11].

In this context, Matrix-Assisted Laser Desorption/Ionization Time-of-Flight Mass Spectrometry (MALDI-TOF MS) has emerged as a revolutionary alternative, allowing microorganism identification in minutes and with low operational cost [14, 15]. In the Brazilian scenario, this technology has already been incorporated by several Central Public Health Laboratories (LACEN), which compose the National MALDI-TOF Identification Network, strengthening the country’s laboratory response capacity [16]. However, although already established for the identification of bacteria and yeasts, the application of MALDI-TOF MS to filamentous and dimorphic fungi presents technical challenges, mainly due to phenotypic variability and cell wall robustness. A critical barrier to identification is the scarcity of representative reference spectra in commercial libraries. These libraries frequently fail in the identification of endemic or genetically diverse species, such as *S. brasiliensis* [17–21].

Previous studies have demonstrated the potential of in-house databases to overcome these commercial limitations and improve the accuracy of species identification within the genus *Sporothrix*. However, most protocols still prioritize the yeast phase for obtaining high-quality spectra, maintaining a dependence on time-consuming subculture steps [18, 22]. Aiming to bridge this technological and care gap in the North region of Brazil, this study aimed to develop and validate an *in-house* spectral library for *S. brasiliensis* composed of isolates originating from the Amazon region of Pará. In addition to ensuring accurate taxonomic identification, we investigated the technical feasibility of direct identification from the filamentous phase (primary growth). By doing so, we sought to eliminate the need for phase conversion and drastically reduce the time to definitive diagnosis.

## Material and methods

### Experimental design and selected isolates

The creation and analysis of the internal MALDI-TOF MS database were performed using a MALDI Biotyper^®^ sirius System (Bruker Daltonics, Bremen, Germany). The first stage of the work consisted of composing the internal database, where 24 clinical isolates of *Sporothrix brasiliensis* were selected (Table 1). The second stage consisted of validating the database, where 46 isolates of the same species were selected (Supplementary Material 1). The selection criterion was that the isolates should originate from human and feline samples and previously characterized by partial sequencing of the calmodulin gene.

**Table 1:** Data regarding the isolates used for the construction of the in-house library

Isolate	Organism	Host	Geography	Genbank
IEC7295	*Sporothrix brasiliensis*	Human	Pará, Brazil	PZ028648
IEC7361	*Sporothrix brasiliensis*	Human	Pará, Brazil	PZ028659
IEC7371	*Sporothrix brasiliensis*	Human	Pará, Brazil	PZ028664
IEC7385	*Sporothrix brasiliensis*	Human	Pará, Brazil	PZ028669
IEC7393	*Sporothrix brasiliensis*	Human	Pará, Brazil	PZ028674
IEC7394	*Sporothrix brasiliensis*	Human	Pará, Brazil	PZ028675
IEC7401	*Sporothrix brasiliensis*	Human	Pará, Brazil	PZ028678
IEC7406	*Sporothrix brasiliensis*	Human	Pará, Brazil	PZ028679
IEC7410	*Sporothrix brasiliensis*	Human	Pará, Brazil	PZ028680
IEC7413	*Sporothrix brasiliensis*	Human	Pará, Brazil	PZ028681
IEC7415	*Sporothrix brasiliensis*	Human	Pará, Brazil	PZ028682
IEC7422	*Sporothrix brasiliensis*	Human	Pará, Brazil	PZ028684
UFRA01	*Sporothrix brasiliensis*	Feline	Pará, Brazil	PZ067653
UFRA02	*Sporothrix brasiliensis*	Feline	Pará, Brazil	PZ067654
UFRA05.1	*Sporothrix brasiliensis*	Feline	Pará, Brazil	PZ067657
UFRA05.2	*Sporothrix brasiliensis*	Feline	Pará, Brazil	PZ067658
UFRA07.2	*Sporothrix brasiliensis*	Feline	Pará, Brazil	PZ067660
UFRA07.3	*Sporothrix brasiliensis*	Feline	Pará, Brazil	PZ067661
UFRA07.4	*Sporothrix brasiliensis*	Feline	Pará, Brazil	PZ067662
UFRA08	*Sporothrix brasiliensis*	Feline	Pará, Brazil	PZ067663
UFRA09.1	*Sporothrix brasiliensis*	Feline	Pará, Brazil	PZ067664
UFRA12.1	*Sporothrix brasiliensis*	Feline	Pará, Brazil	PZ067669
UFRA13.2	*Sporothrix brasiliensis*	Feline	Pará, Brazil	PZ067672
UFRA17	*Sporothrix brasiliensis*	Feline	Pará, Brazil	PZ067680

**Fig. 1:**
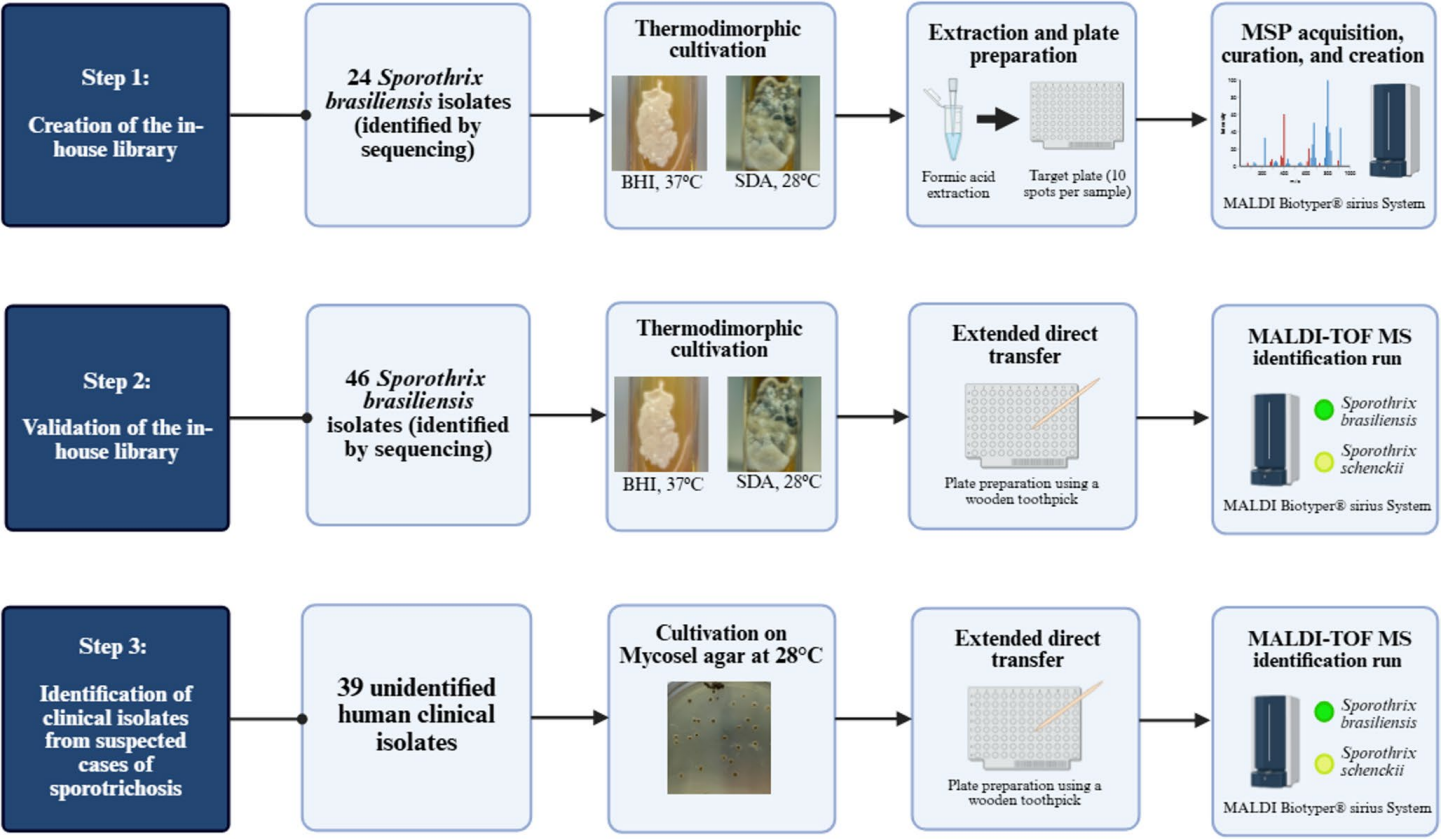
Experimental design flowchart of the study. The process was divided into three main steps: (1) creation of the in-house customized library using 24 *Sporothrix brasiliensis* isolates; (2) validation of the library with 46 isolates previously identified by sequencing; and (3) application of the method for the identification of 39 human clinical isolates from suspected sporotrichosis cases via MALDI-TOF MS

The isolates were analyzed in both morphological phases, exploiting the fungal dimorphism of *S. brasiliensis*. All isolates used for the creation and validation of the in-house library were obtained from the culture collection of the Medical Mycology Laboratory of the Bacteriology and Mycology Section of the Evandro Chagas Institute (IEC/SEBAC/LBMICOL), located in Ananindeua, Pará, Brazil. They are stored in Sabouraud agar culture medium and maintained at room temperature (28℃). The third stage of the work consisted of applying the in-house library to the clinical routine for the identification of 39 isolates from cases suggestive of sporotrichosis (Fig. 1). Detailed descriptions of the specific experimental protocols and procedures are provided in the subsequent subsections.

### Creation of the in-house library

For the yeast phase, a total of 24 Sporothrix brasiliensis isolates were cultured on Brain Heart Infusion (BHI) agar at 37 °C. The process included primary incubation for 7 days, followed by subculturing on new BHI medium for an additional 7 days. For the filamentous phase, the same 24 isolates were cultured on Sabouraud Dextrose agar and incubated at 28 °C for 7 days. Regardless of the morphology, the cellular biomass was processed according to the standard tube extraction protocol recommended by Bruker [23]. For morphological confirmation, microscopic characteristics of both phases were observed under an optical microscope (40× objective lens) after staining with Lactophenol Cotton Blue.

The colonies were suspended in 300 µL of HPLC-grade water and 900 µL of absolute ethanol. The mixture was centrifuged at 13,000 rpm for 2 min. After discarding the supernatant and completely removing the residual ethanol, the pellet was resuspended in 50 µL of 70% formic acid and 50 µL of acetonitrile. It was then centrifuged again under the same conditions. Finally, 1 µL of the supernatant was applied to the target plate, dried at room temperature, and overlaid with 1 µL of HCCA matrix for subsequent mass spectrometry analysis.

The construction of the library followed the standardized protocols by Bruker [23]. Ten replicates of each isolate were deposited on the target plate. A spot containing *Bacterial Test Standard* (BTS) was included in each analytical run for instrument calibration. The samples were analyzed in the FlexControl software (v.3.4), resulting in 30 spectra per sample (triplicate per spot). The processing of the raw spectra, including smoothing and baseline subtraction, was performed in FlexAnalysis (v.3.4). After visual inspection for artifact removal and confirmation of peak tolerance (< 500 ppm), a minimum set of 20 spectra was used to compose each entry.

The MBT Compass Explorer software was used both for the creation of the Main Spectrum Profiles (MSPs) and for the generation of the similarity analysis dendrogram between the created MSPs and in relation to the MSPs in Bruker’s commercial library. In the MSP dendrogram, a distance level of zero indicates total similarity, and 1000 means complete dissimilarity.

### Validation of the in-house library

The cultivation conditions for validation followed the same protocols described for the construction of the in-house library (Sect.  2.2), using Sabouraud Dextrose agar at 28 °C for 7 days for the filamentous phase and BHI agar at 37 °C for the yeast phase. Sample preparation followed the extended direct transfer protocol recommended by the manufacturer. With the aid of a sterile wooden toothpick, a small amount of fungal biomass was collected and spread on the target plate with subsequent addition of 1 µL of formic acid (70%) onto each sample. After drying at room temperature, each spot was overlaid with 1 µL of HCCA matrix solution and kept at room temperature until final crystallization, for later analysis in the equipment. MALDI-TOF MS identification was performed using one identification round with the MSP reference libraries provided by Bruker, along with the in-house database developed in this study. The identification scores were evaluated following Bruker’s standard recommended criteria, where a score value ≥ 2.0 is considered reliable for species-level identification, and a score value ≥ 1.7 is acceptable for genus-level identification.

### Identification of clinical isolates from suspected cases of sporotrichosis

After validation of the in-house database, the library was employed to identify isolates obtained from routine culture clinical samples. The material collected from the lesion, using a swab, was initially stored in BHI broth until processing at IEC/SEBAC/LBMICOL. In the laboratory, the samples were centrifuged and the resulting pellet was inoculated onto Mycosel agar, followed by incubation at 28 °C. Following fungal growth with characteristic *Sporothrix* genus morphology, which ranged from 5 to 7 days, the mycelium of the colony was removed with the aid of a wooden stick previously immersed in 70% formic acid and applied to a spot on the polished steel MALDI plate. 1 µL of 70% formic acid was added to each spot containing the sample. After drying at room temperature, 1 µL of HCCA matrix was deposited onto the sample and again left to dry before analysis.

### Data analysis

Data tabulation and descriptive statistical analyses were performed using the R language, through the integrated development environment RStudio (version 2025.09.02 + 418). The library’s performance was evaluated through measures of central tendency and dispersion (interquartile range) of the *Score* values obtained. Graph creation for visualizing the distribution of scores was conducted using the *ggplot2* package.

## Results

**Fig. 2:**
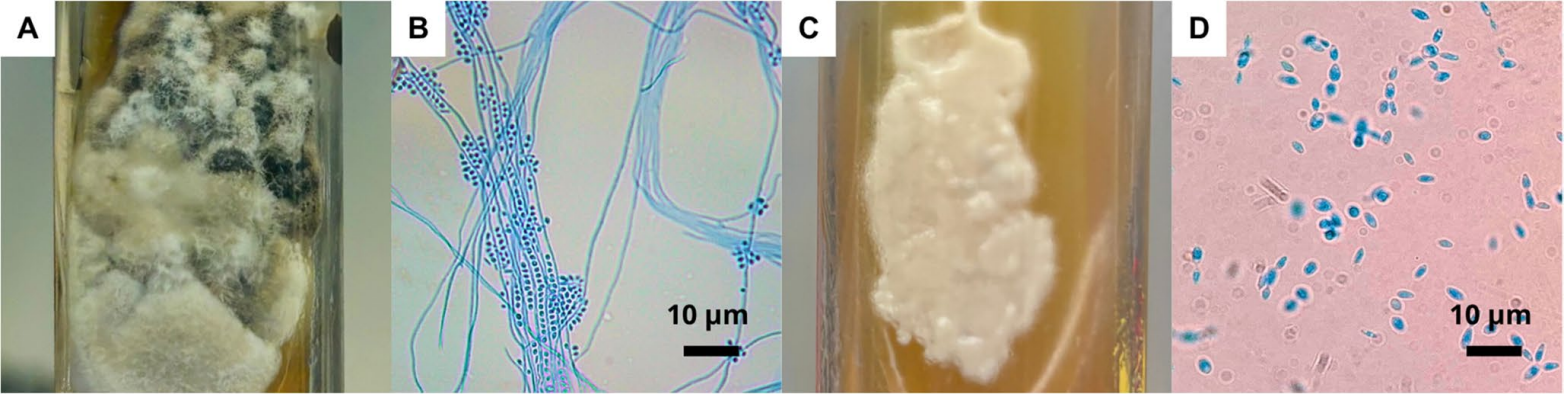
Macroscopic and micromorphological aspects of *Sporothrix brasiliensis*. (**A**) Macromorphology of the filamentous phase cultured on SDA at 28 °C. (**B**) Micromorphology of the filamentous phase stained with Lactophenol Cotton Blue and observed under a 40× objective lens. (**C**) Macromorphology of the yeast-like phase cultured on BHI agar at 37 °C. (**D**) Micromorphology of the yeast-like phase stained with Lactophenol Cotton Blue and observed under a 40× objective lens

For the construction of the reference database, 37 main spectral profiles were successfully generated, with 24 MSPs representative of the yeast-like form and 13 MSPs representative of the filamentous form of the species *Sporothrix brasiliensis* (Fig. 2). Although all 24 isolates were analyzed in both morphological phases, 11 isolates from the filamentous phase were unable to generate MSPs because they failed to meet the strict spectral quality and reproducibility thresholds required by the software, exhibiting high baseline noise and spectral instability inherent to this morphology.

**Fig. 3:**
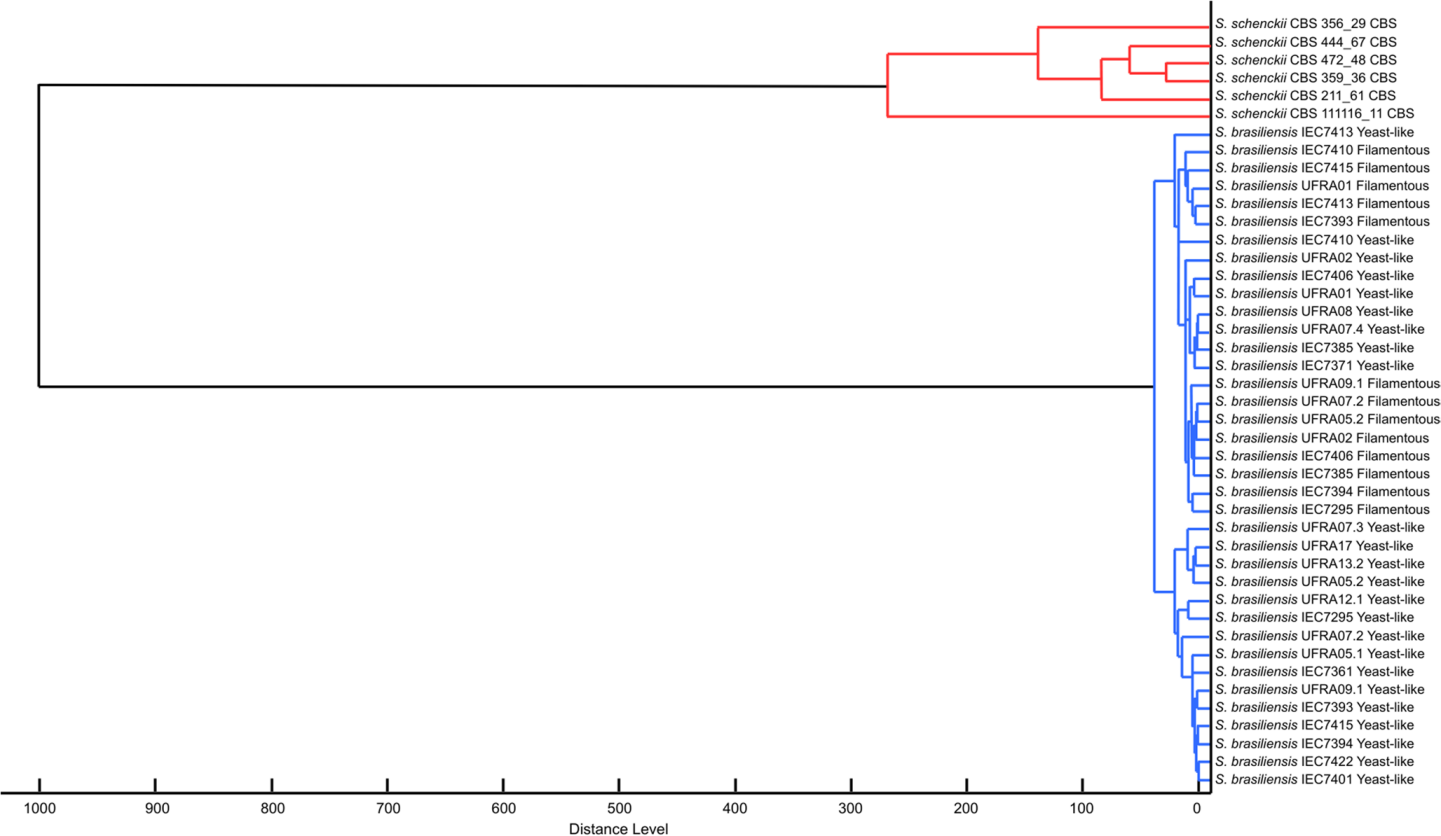
Dendrogram of *Sporothrix* species main spectrum profiles (MSPs). The analysis includes a total of 43 profiles: 37 in-house *Sporothrix brasiliensis* MSPs (indicated by blue branches, comprising 24 in the yeast-like phase and 13 in the filamentous phase) and 6 *S. schenckii* reference MSPs from the commercial database (indicated by red branches). A distance level of zero indicates total similarity, and 1000 means complete dissimilarity

Upon confronting the protein profiles obtained in this study with reference MSPs of *Sporothrix schenckii* from the commercial *Filamentous Fungi* library (Bruker Daltonics) through the MSP Dendrogram analysis, the formation of two distinct main clades was observed at a distance level of 1000 (Fig. 3).

**Fig. 4:**
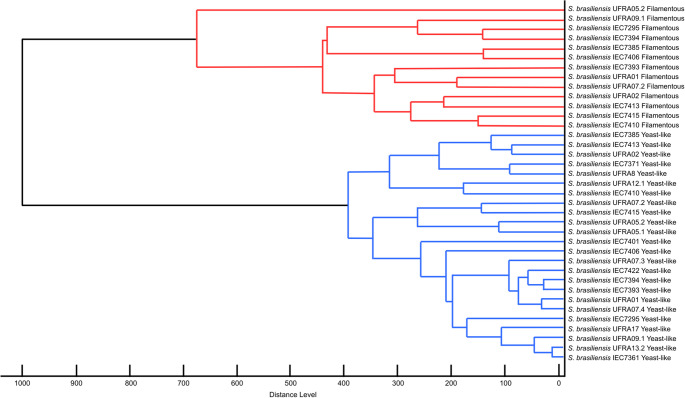
Dendrogram of 37 *Sporothrix brasiliensis* main spectrum profiles (MSPs) created for the in-house library. Blue branches correspond to MSPs from the yeast form (*n* = 24) and red branches to MSPs from the filamentous form (*n* = 13)

The in-house library’s internal structure was evaluated using a dendrogram containing exclusively the created MSPs (Fig. 4). The analysis revealed a separation based on fungal dimorphism, with the formation of two main clades at a distance level of 1000.

**Fig. 5:**
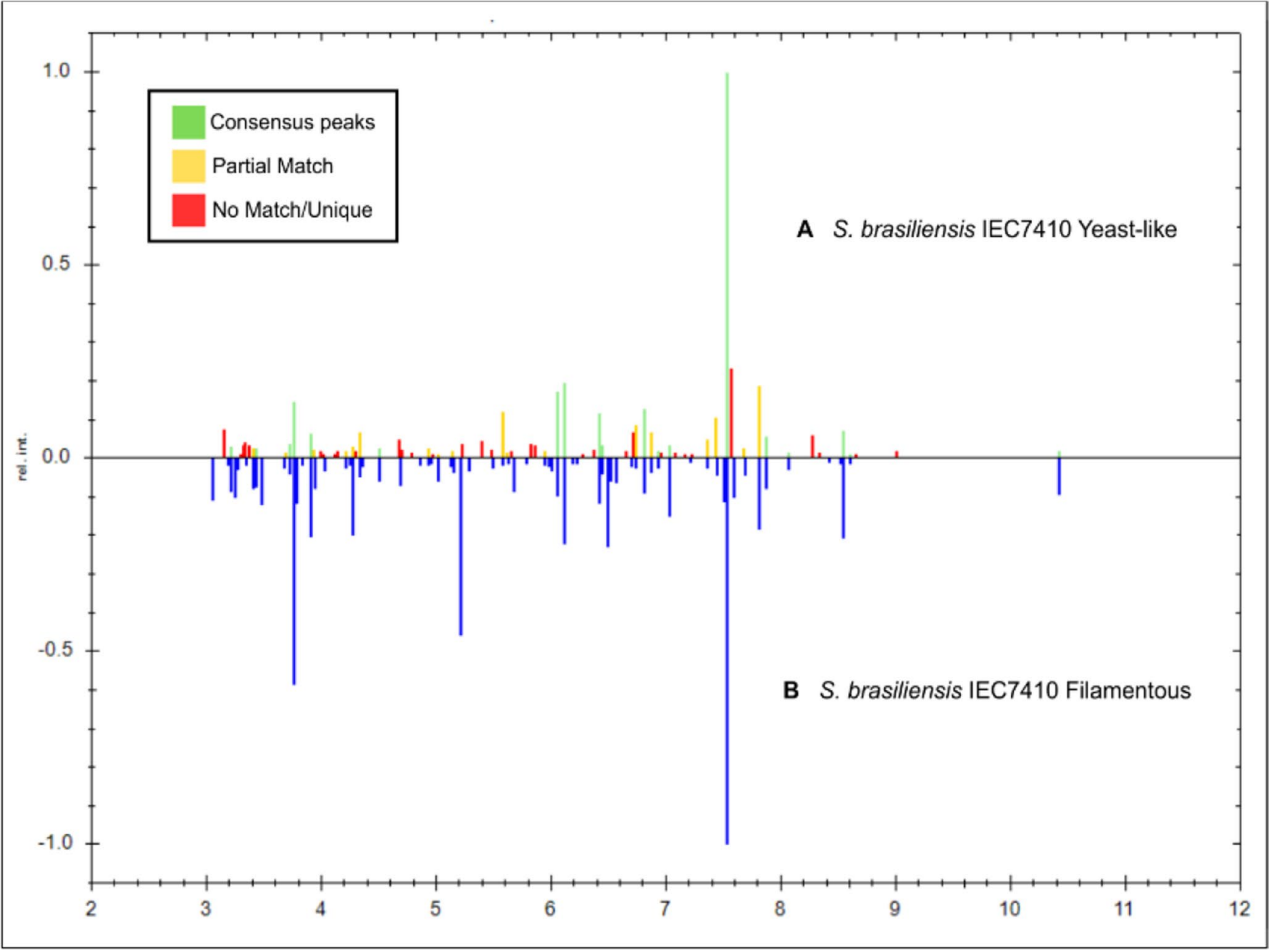
Comparison of two Main Spectra Profiles (MSPs). The upper spectrum (**A**) represents IEC7410 yeast-like MSP and the lower (**B**), inverted spectrum represents IEC7410 filamentous MSP. Green signals indicate common peaks shared by both profiles, yellow signals represent minor matching peaks, and red signals indicate unique peaks found only in the respective spectrum

The observed separation in the dendrogram is a direct reflection of marked and consistent differences in the spectral profiles of each morphology. Figure 5 illustrates this point in detail, comparing the spectra of the yeast and filamentous forms for a representative strain from the database, IEC7410.

Comparative analysis of the MSPs revealed distinct, yet complementary, protein signatures for the yeast and filamentous phases of *S. brasiliensis* (Fig. 5). The mirror comparison evidenced the presence of conserved high-intensity peaks, with a highlight on the biomarker at 7530 m/z, consistently identified in both morphologies. However, prominent variations were observed in the intensity and presence of ions in the range of 2000 to 12,000 m/z. Notably, a trend of differential expression of biomarkers was observed in regions near 5500 m/z and 8200 m/z, which appear to contribute to the spectral segregation between the morphological phases observed in the dendrogram. Spectra confirming the consistent presence of these peaks from all *S. brasiliensis* strains used for library development are provided in the Supplementary Material 1.

**Fig. 6:**
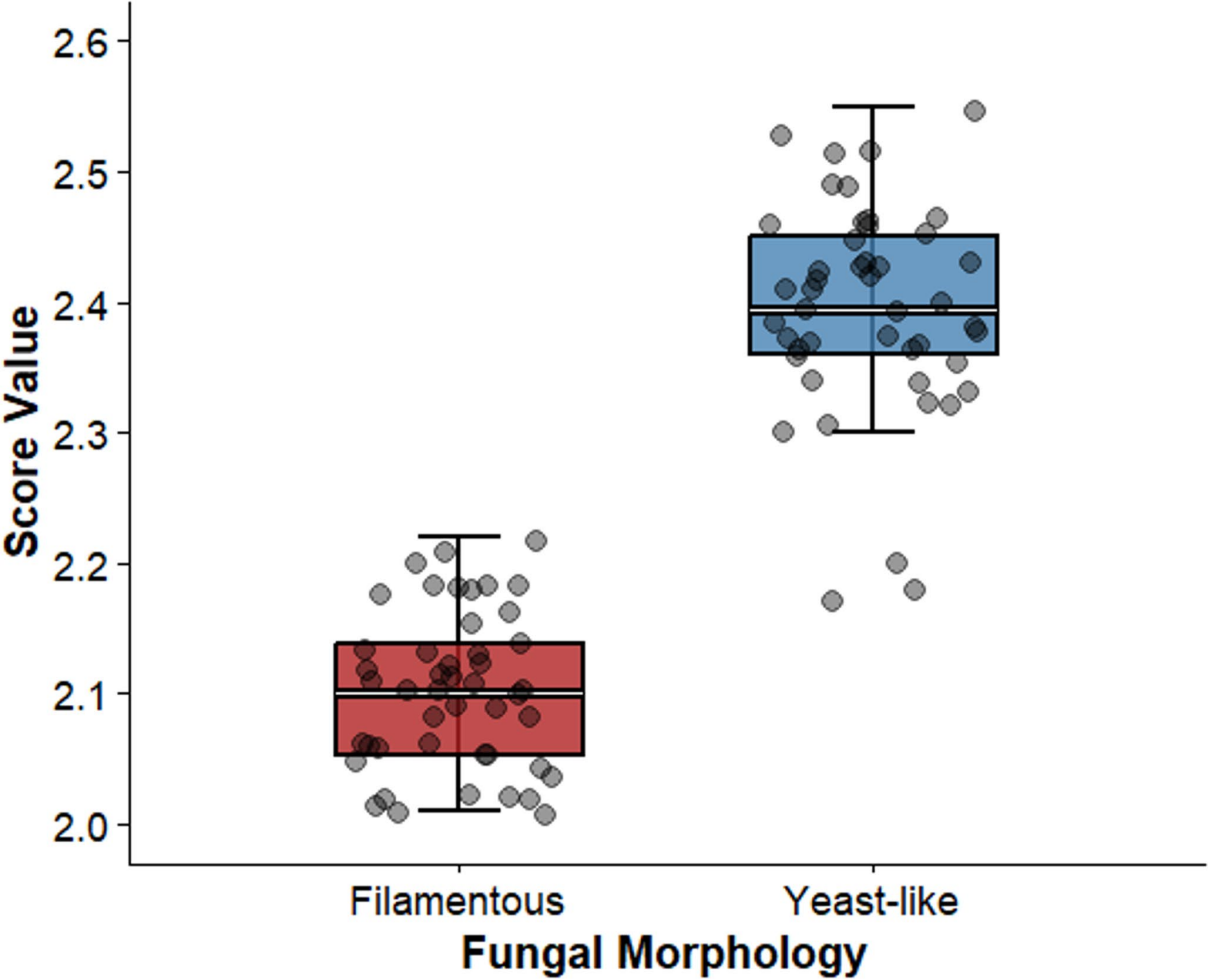
MALDI-TOF scores distribution according to fungal morphology. Comparison between *Filamentous* and *Yeast-like* phases (*n* = 46). The data demonstrate consistently higher scores for the yeast phase. The box plot indicates the median, quartiles, and individual values. All scores are within the reliable species-level identification range

The accuracy validation results confirm the high performance of the *in-house* library in identifying *Sporothrix brasiliensis* at the species level, regardless of the analyzed morphofunctional phase (Fig. 6). By adopting a score threshold greater than or equal to 2.0, it was observed that 100% of the isolates were correctly identified with high confidence indices, which confirms the diagnostic precision of the developed database. The analysis of the distribution of these scores shows that the yeast-like phase (*Yeast-like*) presents superior analytical robustness to the filamentous phase (*Filamentous*). Although both morphologies consistently exceeded the species identification threshold, the yeast-like phase stood out for presenting a higher median score and a lower interquartile dispersion. This profile indicates that the yeast morphology generates more stable and reproducible protein profiles in the MALDI-TOF system, optimizing the performance of the developed library.

**Fig. 7:**
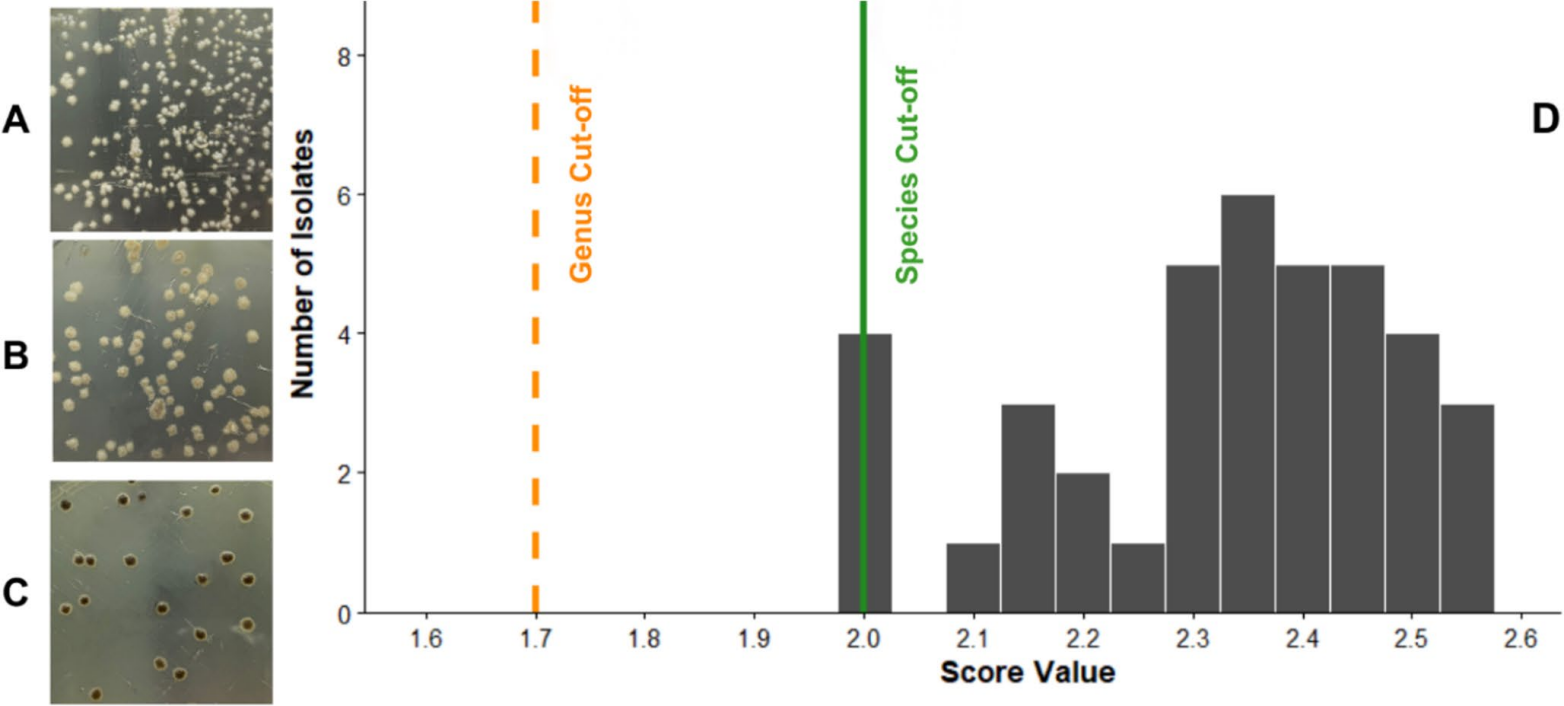
Application of the In-House library with clinical routine isolates. Panels **A**, **B**, and **C**: Representative images of the macroscopic aspect of cultures of three clinical isolates obtained from lesion swabs. Panel **D**: Histogram of the score value distribution for the 39 tested isolates

In a third stage, to evaluate the applicability of the created library in a routine diagnostic scenario, it was tested against 39 clinical isolates recovered from primary growth from patients. Notably, all isolates were correctly identified as *S. brasiliensis*, presenting scores greater than 2.0 (Fig. 7).

## Discussion

*Sporothrix. brasiliensis* sporotrichosis represents a critical public health challenge in Brazil, with accelerated geographic expansion and recent compulsory notification [3, 4, 12]. This scenario is particularly worrying in the North region, where reports are still scarce when compared to the hyperendemic areas of the South and Southeast [9]. Although clinical management is started early based on diagnostic suspicion, species-level identification remains fundamental for epidemiological monitoring and understanding. However, while molecular methods have high costs and longer turnaround times, MALDI-TOF MS technology allows identification in a few minutes [11, 13, 24]. In Brazil, this technique is already integrated into the National Identification Network through LACENs and reference centers [16]. Therefore, by validating a library with local strains, this study offers a fast and cost-effective alternative to overcome regional diagnostic limitations, enabling the real mapping of *S. brasiliensis* circulation in the Brazilian Amazon region.

Despite its potential, the application of MALDI-TOF MS for thermodimorphic fungi faces significant technical obstacles [20, 25]. Commercial databases, such as Bruker, often restrict taxonomic resolution to the genus level for *Sporothrix* and *Talaromyces* species, mainly due to the scarcity of representative spectra [25]. To mitigate this gap, the development of in-house databases has proven effective, with documented success in the identification of *Paracoccidioides lutzii*, *Paracoccidioides brasiliensis*, and *Histoplasma capsulatum* [26, 27]. Following this approach, Oliveira et al. [18]. demonstrated that a comprehensive, molecularly validated, and geographically broad spectral library allows for the accurate identification of *Sporothrix* species.

In our study, the construction of the library resulted in 24 main spectrum profiles (MSPs) for the yeast-like phase and 13 for the filamentous phase. This variation reflects the rigorous quality criterion required to create stable reference profiles. The mycelial morphology is inherently more heterogeneous than the yeast-like morphology, being composed of a complex network of hyphae and conidia which makes it difficult to obtain highly reproducible spectra [28, 29]. Consequently, we prioritized the inclusion of a more restricted number of filamentous MSPs that achieved superior internal correlation indices and lower baseline noise, ensuring the robustness of the database to the detriment of the volumetric quantity of entries.

A central point in the identification of dimorphic fungi is phenotypic variation. Our results revealed distinct mass profiles between the mycelial and yeast phases, a distinction likely attributable to phase-dependent differential gene expression [22]. This divergence was confirmed by the segregation of the phases into independent clades in our dendrogram. As this spectral difference can induce diagnostic errors depending on the cultured morphology, the implementation of a mixed library (yeast and filament) is essential to increase the robustness of the system.

In the validation, the yeast phase confirmed itself as the gold standard [18], with 100% accuracy and high *score* values. However, the differential of this study lies in the performance of the mycelial phase. Contrary to reports of spectral overlap that would limit identification [22], our library correctly identified 100% of the clinical isolates from filamentous growth. The analytical disparity between the phases, with higher *scores* in the yeast phase, is attributed to the greater ease of cell lysis and homogeneity of this phase [18]. In contrast, the more robust cell wall of the filamentous phase reduces the efficiency of protein extraction by the direct transfer protocol [28, 29]. This advance expands on what was observed for *S. schenckii* and *S. globosa* [17], proving that early identification in the mycelial phase is viable and accurate also for *S. brasiliensis*. Such a finding is crucial, given that the commercial library available in the system was not capable of identifying isolates of the *S. brasiliensis* species, reinforcing the need to build local banks that cover the main species of zoonotic sporotrichosis occurring in the country [3].

The optimization of the workflow represents the most relevant practical impact of this work. While previous protocols required subcultures in BHI medium, long incubation periods, and complex adjustments to the cut-offs [18, 22], the construction of the in-house library allowed for the direct identification of primary growth isolates. This approach eliminated the need for morphological conversion, accelerating diagnosis from weeks to just 5 to 7 days. Although initial clinical management may occur empirically, the speed of this method is crucial for laboratory surveillance. In the current epidemiological scenario in Pará (northern region of Brazil), where *S. brasiliensis* shows rapid expansion [9], the ability to process a large volume of samples with low operational cost and without the complexity of molecular methods transforms the reference laboratory into an efficient real-time monitoring unit. This speed in species identification is critical data for outbreak management, allowing health authorities to map the dispersion of the pathogen and plan One Health interventions much earlier than would be possible through conventional culture methods.

Despite the demonstrated high diagnostic accuracy, this study presents limitations that should be considered. Firstly, the development and validation of the *in-house* library were conducted in a single-center study, using isolates primarily from a specific geographic area (Pará/Northern Brazil). Considering that *S. brasiliensis* exhibits documented genetic variability [30], the future inclusion of strains from other endemic regions, both within the country and abroad, would be ideal to ensure the global representativeness of the database.

Secondly, the scope of the library prioritized *S. brasiliensis* due to its current epidemiological relevance, leaving the inclusion of other complex agents, such as *S. globosa* and *S. luriei*, as a future study. Additionally, the representativeness of the database is restricted to clinical isolates obtained from human and feline hosts. The absence of environmental isolates currently prevents the confirmation of the tool’s applicability for environmental surveillance, as it was not possible to evaluate variations in the spectral profile of saprobic strains. Finally, although we have demonstrated success in direct identification from the filamentous phase, the multicentric standardization of this protocol is necessary to attest to its interlaboratory reproducibility across different mass spectrometry platforms available in the country.

## Conclusion

The in-house developed library overcomes the limitations of commercial databases, establishing MALDI-TOF MS (Bruker) as a robust tool for the identification of *S. brasiliensis*. The main breakthrough of this study was demonstrating the viability of direct diagnosis from filamentous primary growth, eliminating the need for time-consuming conversion steps to the yeast phase. Thus, we validate a rapid, accurate, and technologically advanced solution applicable to both human and feline samples, ready to optimize the diagnostic routine in our region for those who use the Bruker platform for *S. brasiliensis* identification.

## Supplementary Information

Below is the link to the electronic supplementary material.

Supplementary Material 1 (DOCX 738 KB)
