# Supplementary material for: Rapid identification of Sporothrix brasiliensis by MALDI-TOF MS directly from clinical cultures in an Amazonian epidemic setting
Source: Braz J Microbiol. 2026 Jul 1;57(1):186. doi: 10.1007/s42770-026-01996-8 (PMC13323457; doi:10.1007/s42770-026-01996-8)
Supplement: Supplementary file 1 — Supplementary Material 1 (DOCX 738 KB) [file 42770_2026_1996_MOESM1_ESM.docx]

**Rapid identification of *Sporothrix brasiliensis* by MALDI-TOF MS directly from clinical cultures in an Amazonian epidemic setting**

**Brazilian Journal of Microbiology**

Daniel dos Santos Caldas^1*^, Gabriel Silas Marinho de Sousa^1^, Pedro Henrique Oliveira Favacho^1^, Rodrigo Santos de Oliveira^2^, Elaine Patrícia Tavares do Espírito Santo^2^ e Silvia Helena Marques da Silva^1,2^

^1^ Programa de Pós-Graduação em Biologia de Agentes Infecciosos e Parasitários, Instituto de Ciências Biológicas, Universidade Federal do Pará, Belém 66075-110, Pará, Brazil

^2^ Laboratório de Micoses Superficiais e Sistêmicas, Seção de Bacteriologia e Micologia, Instituto Evandro Chagas, Ananindeua 67030-000, Pará, Brazil

*Corresponding author email: [dancaldas@yahoo.com](mailto:dancaldas@yahoo.com)

**SUPPLEMENTARY MATERIAL**

**Table S1** Data regarding the isolates used for validation of the in-house library

| **Isolate** | **Source** | **Organism** | **Geography** | **Genbank** | **MALDI TOF MS Score** | |
| --- | --- | --- | --- | --- | --- | --- |
|  |  |  |  |  | **Filamentous** | **Yeast-like** |
| IEC7321 | Human | *Sporothrix brasiliensis* | Pará, Brazil | PZ028649 | 2.01 | 2.39 |
| IEC7322 | Human | *Sporothrix brasiliensis* | Pará, Brazil | PZ028650 | 2.12 | 2.31 |
| IEC7325 | Human | *Sporothrix brasiliensis* | Pará, Brazil | PZ028651 | 2.08 | 2.33 |
| IEC7332 | Human | *Sporothrix brasiliensis* | Pará, Brazil | PZ028652 | 2.12 | 2.46 |
| IEC7334 | Human | *Sporothrix brasiliensis* | Pará, Brazil | PZ028653 | 2.10 | 2.42 |
| IEC7345 | Human | *Sporothrix brasiliensis* | Pará, Brazil | PZ028654 | 2.11 | 2.39 |
| IEC7346 | Human | *Sporothrix brasiliensis* | Pará, Brazil | PZ028655 | 2.02 | 2.46 |
| IEC7347 | Human | *Sporothrix brasiliensis* | Pará, Brazil | PZ028656 | 2.10 | 2.43 |
| IEC7348 | Human | *Sporothrix brasiliensis* | Pará, Brazil | PZ028657 | 2.10 | 2.36 |
| IEC7350 | Human | *Sporothrix brasiliensis* | Pará, Brazil | PZ028658 | 2.18 | 2.37 |
| IEC7363 | Human | *Sporothrix brasiliensis* | Pará, Brazil | PZ028660 | 2.06 | 2.35 |
| IEC7392 | Human | *Sporothrix brasiliensis* | Pará, Brazil | PZ028673 | 2.04 | 2.41 |
| IEC7397 | Human | *Sporothrix brasiliensis* | Pará, Brazil | PZ028676 | 2.01 | 2.18 |
| IEC7399 | Human | *Sporothrix brasiliensis* | Pará, Brazil | PZ028677 | 2.16 | 2.20 |
| IEC7417 | Human | *Sporothrix brasiliensis* | Pará, Brazil | PZ028683 | 2.05 | 2.17 |
| IEC7425 | Human | *Sporothrix brasiliensis* | Pará, Brazil | PZ028686 | 2.18 | 2.55 |
| IEC7428 | Human | *Sporothrix brasiliensis* | Pará, Brazil | PZ028687 | 2.14 | 2.37 |
| IEC7430 | Human | *Sporothrix brasiliensis* | Pará, Brazil | PZ028688 | 2.06 | 2.32 |
| IEC7433 | Human | *Sporothrix brasiliensis* | Pará, Brazil | PZ028689 | 2.21 | 2.52 |
| IEC7437 | Human | *Sporothrix brasiliensis* | Pará, Brazil | PZ028690 | 2.13 | 2.38 |
| IEC7439 | Human | *Sporothrix brasiliensis* | Pará, Brazil | PZ028691 | 2.18 | 2.43 |
| IEC7441 | Human | *Sporothrix brasiliensis* | Pará, Brazil | PZ028692 | 2.13 | 2.38 |
| IEC7442 | Human | *Sporothrix brasiliensis* | Pará, Brazil | PZ028693 | 2.11 | 2.34 |
| IEC7534 | Human | *Sporothrix brasiliensis* | Pará, Brazil | PZ028694 | 2.06 | 2.43 |
| IEC7535 | Human | *Sporothrix brasiliensis* | Pará, Brazil | PZ028695 | 2.02 | 2.37 |
| IEC7552 | Human | *Sporothrix brasiliensis* | Pará, Brazil | PZ028702 | 2.18 | 2.46 |
| IEC7553 | Human | *Sporothrix brasiliensis* | Pará, Brazil | PZ028703 | 2.15 | 2.45 |
| UFRA10 | Feline | *Sporothrix brasiliensis* | Pará, Brazil | PZ067666 | 2.22 | 2.36 |
| UFRA11.1 | Feline | *Sporothrix brasiliensis* | Pará, Brazil | PZ067667 | 2.01 | 2.46 |
| UFRA11.2 | Feline | *Sporothrix brasiliensis* | Pará, Brazil | PZ067668 | 2.11 | 2.49 |
| UFRA14 | Feline | *Sporothrix brasiliensis* | Pará, Brazil | PZ067673 | 2.06 | 2.41 |
| UFRA15.2 | Feline | *Sporothrix brasiliensis* | Pará, Brazil | PZ067675 | 2.11 | 2.32 |
| UFRA16.1 | Feline | *Sporothrix brasiliensis* | Pará, Brazil | PZ067678 | 2.12 | 2.43 |
| UFRA16.2 | Feline | *Sporothrix brasiliensis* | Pará, Brazil | PZ067679 | 2.2 | 2.38 |
| UFRA18 | Feline | *Sporothrix brasiliensis* | Pará, Brazil | PZ067681 | 2.02 | 2.49 |
| UFRA19.2 | Feline | *Sporothrix brasiliensis* | Pará, Brazil | PZ067683 | 2.09 | 2.46 |
| UFRA21.1 | Feline | *Sporothrix brasiliensis* | Pará, Brazil | PZ067685 | 2.05 | 2.53 |
| UFRA22.1 | Feline | *Sporothrix brasiliensis* | Pará, Brazil | PZ067687 | 2.05 | 2.42 |
| UFRA22.2 | Feline | *Sporothrix brasiliensis* | Pará, Brazil | PZ067688 | 2.09 | 2.37 |
| UFRA22.3 | Feline | *Sporothrix brasiliensis* | Pará, Brazil | PZ067689 | 2.18 | 2.30 |
| UFRA23.1 | Feline | *Sporothrix brasiliensis* | Pará, Brazil | PZ067690 | 2.1 | 2.51 |
| UFRA23.2 | Feline | *Sporothrix brasiliensis* | Pará, Brazil | PZ067691 | 2.08 | 2.45 |
| UFRA24.1 | Feline | *Sporothrix brasiliensis* | Pará, Brazil | PZ067692 | 2.04 | 2.34 |
| UFRA24.2 | Feline | *Sporothrix brasiliensis* | Pará, Brazil | PZ067693 | 2.18 | 2.40 |
| UFRA25 | Feline | *Sporothrix brasiliensis* | Pará, Brazil | PZ067694 | 2.13 | 2.42 |
| UFRA26.2 | Feline | *Sporothrix brasiliensis* | Pará, Brazil | PZ067695 | 2.02 | 2.36 |


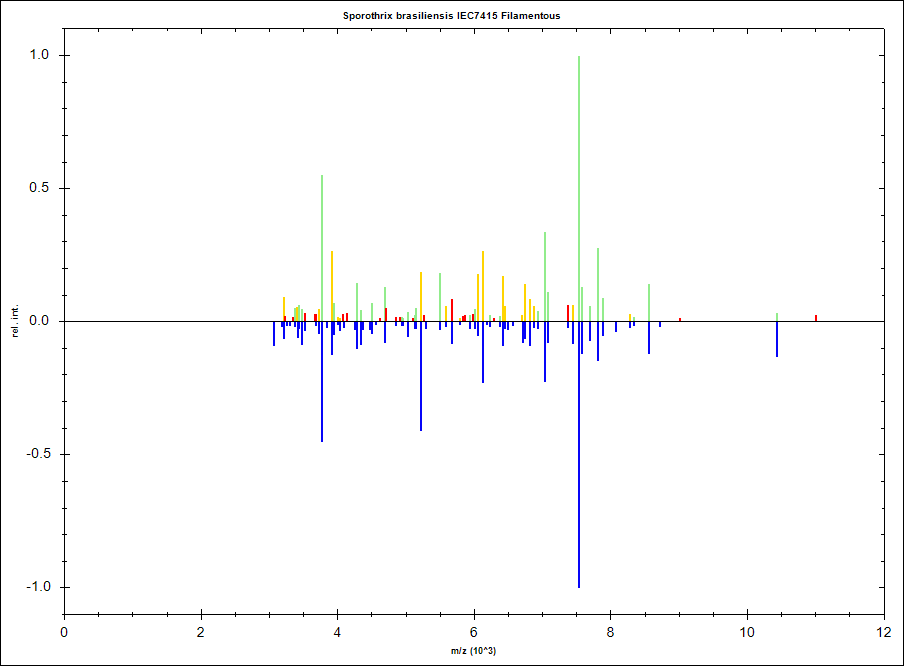


**Fig. S1** Comparison of two Main Spectra Profiles (MSPs). The upper spectrum represents IEC7415 yeast-like MSP and the lower, inverted spectrum represents IEC7415 filamentous MSP


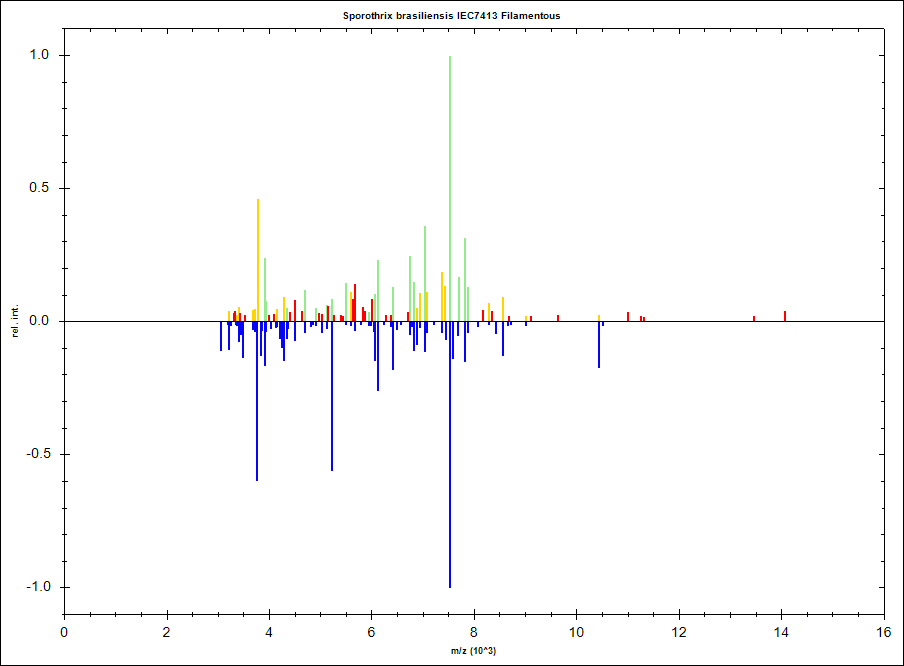


**Fig. S2** Comparison of two Main Spectra Profiles (MSPs). The upper spectrum represents IEC7413 yeast-like MSP and the lower, inverted spectrum represents IEC7413 filamentous MSP


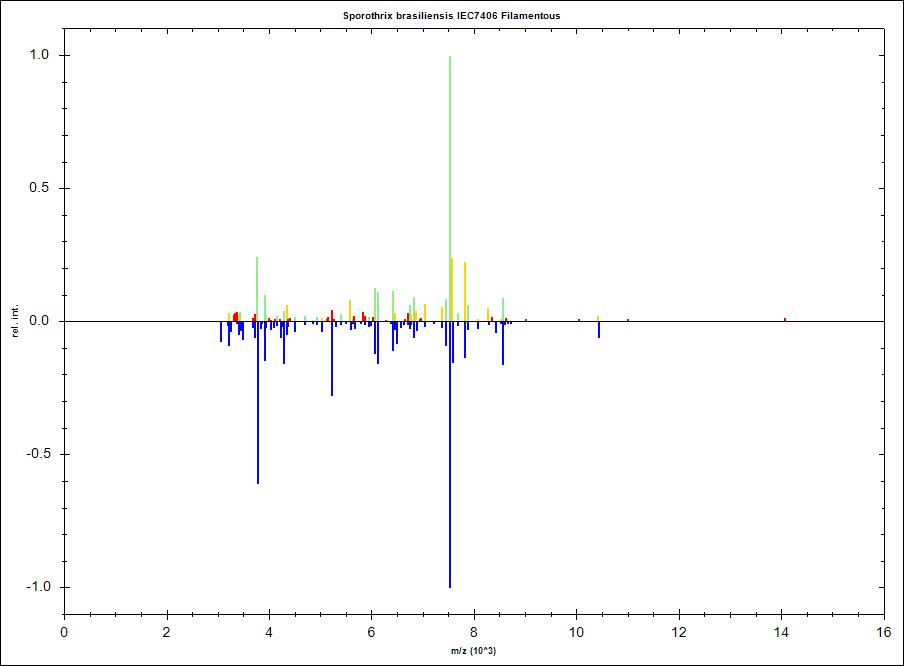


**Fig. S3** Comparison of two Main Spectra Profiles (MSPs). The upper spectrum represents IEC7406 yeast-like MSP and the lower, inverted spectrum represents IEC7406 filamentous MSP


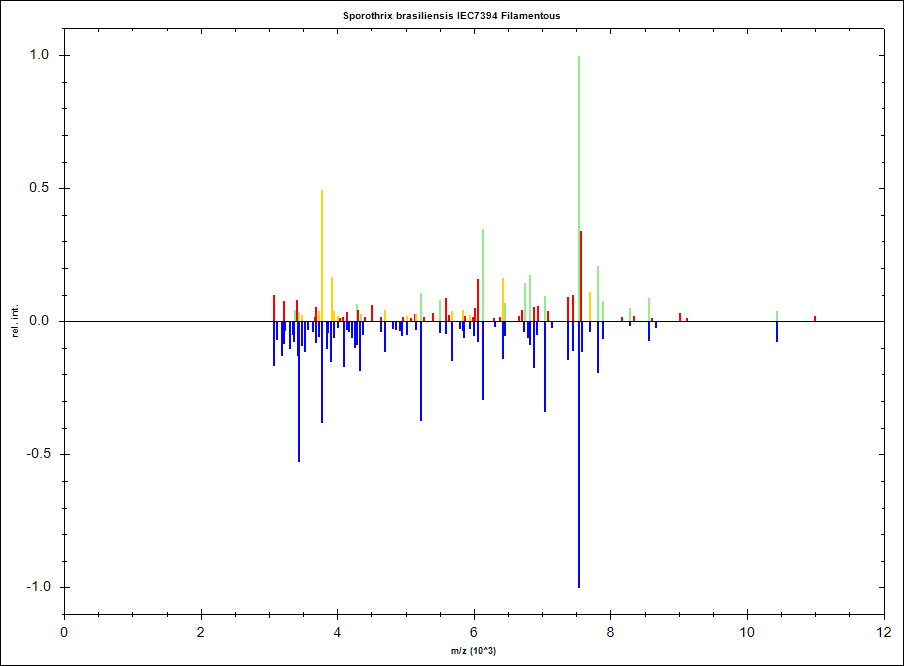


**Fig. S4** Comparison of two Main Spectra Profiles (MSPs). The upper spectrum represents IEC7394 yeast-like MSP and the lower, inverted spectrum represents IEC7394 filamentous MSP


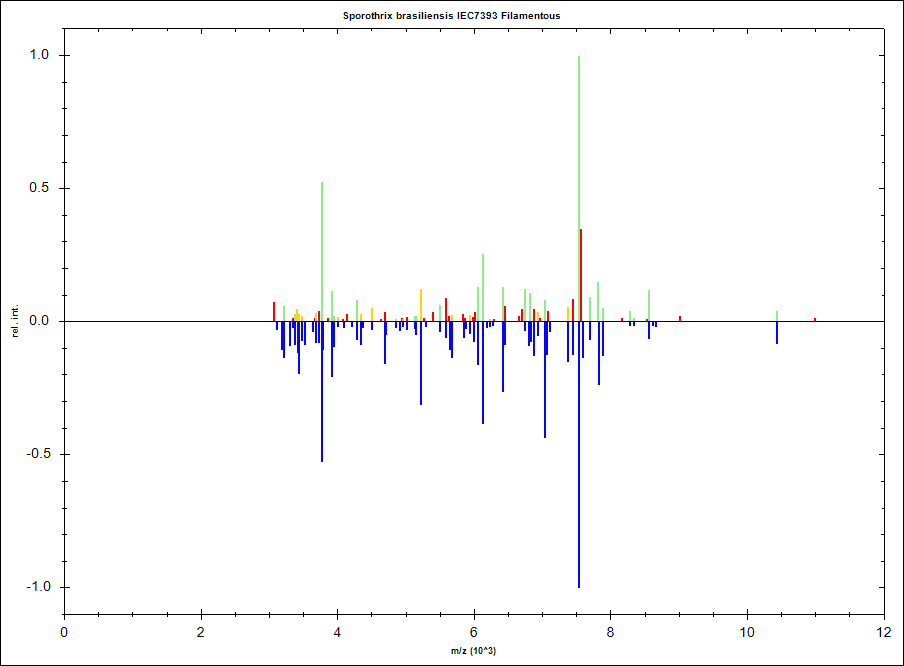


**Fig. S5** Comparison of two Main Spectra Profiles (MSPs). The upper spectrum represents IEC7393 yeast-like MSP and the lower, inverted spectrum represents IEC7393 filamentous MSP


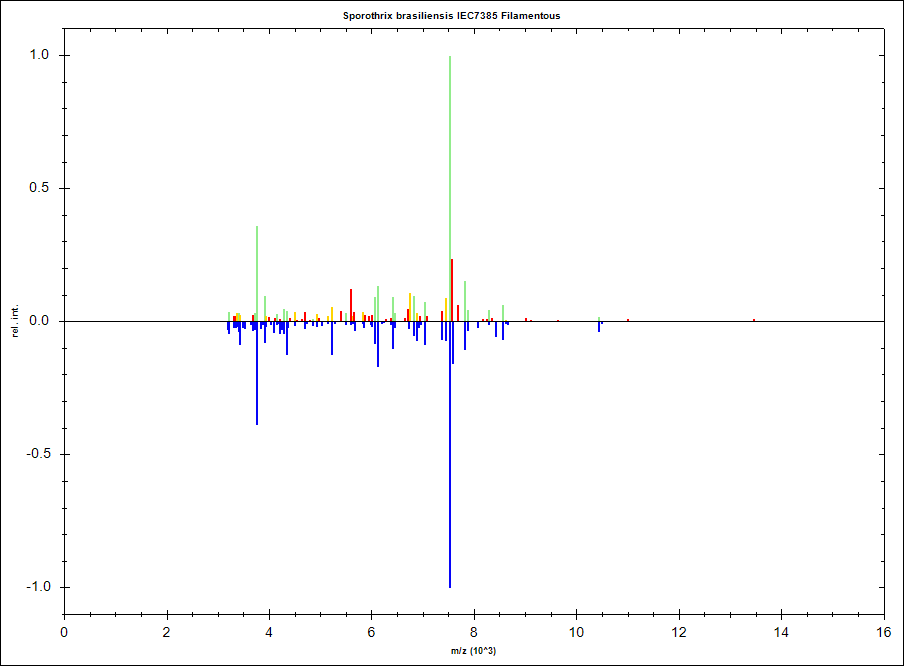


**Fig. S6** Comparison of two Main Spectra Profiles (MSPs). The upper spectrum represents IEC7385 yeast-like MSP and the lower, inverted spectrum represents IEC7385 filamentous MSP


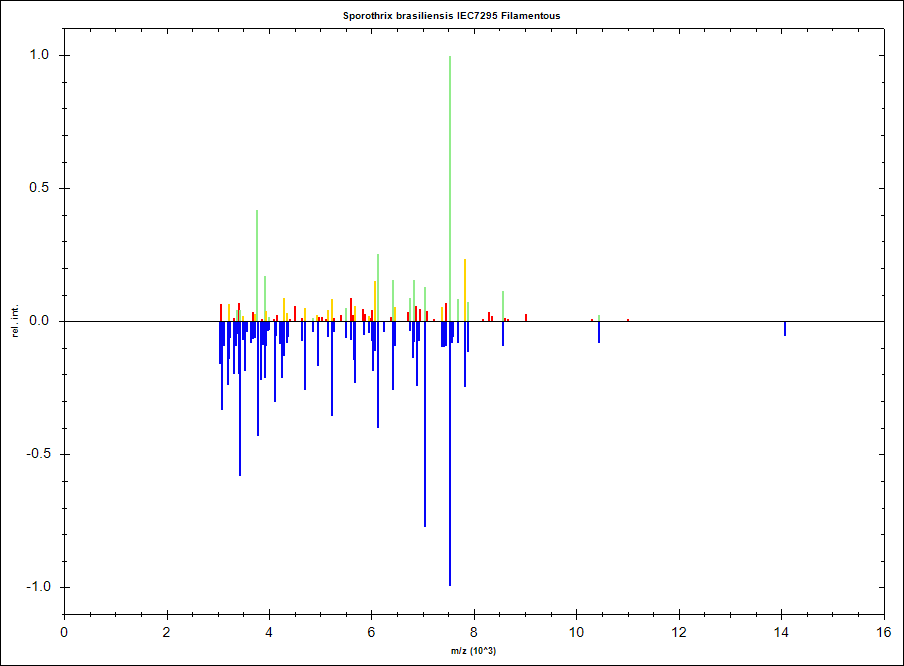


**Fig. S7** Comparison of two Main Spectra Profiles (MSPs). The upper spectrum represents IEC7295 yeast-like MSP and the lower, inverted spectrum represents IEC7295 filamentous MSP


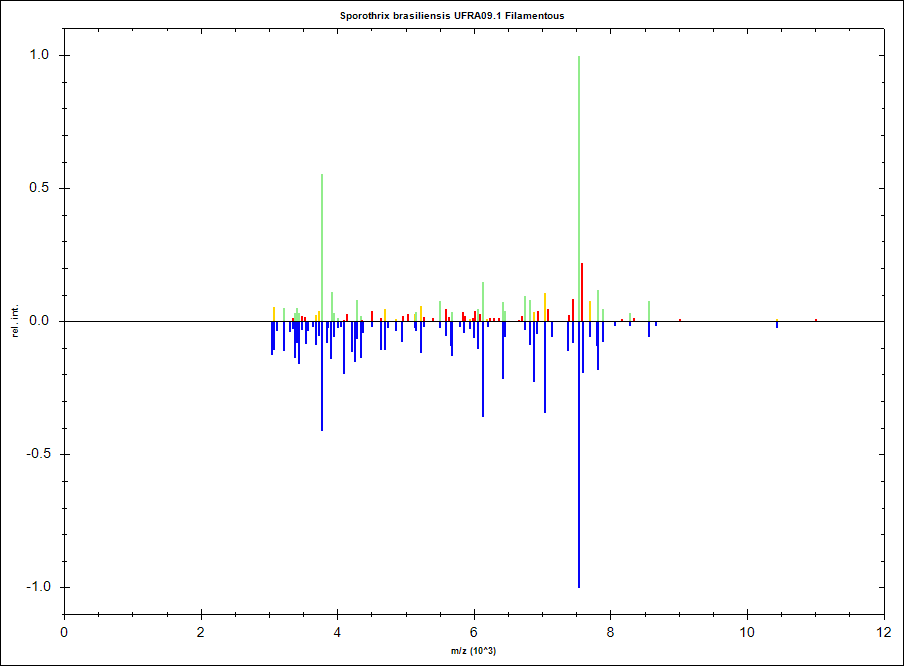


**Fig. S8** Comparison of two Main Spectra Profiles (MSPs). The upper spectrum represents UFRA09.1 yeast-like MSP and the lower, inverted spectrum represents UFRA09.1 filamentous MSP


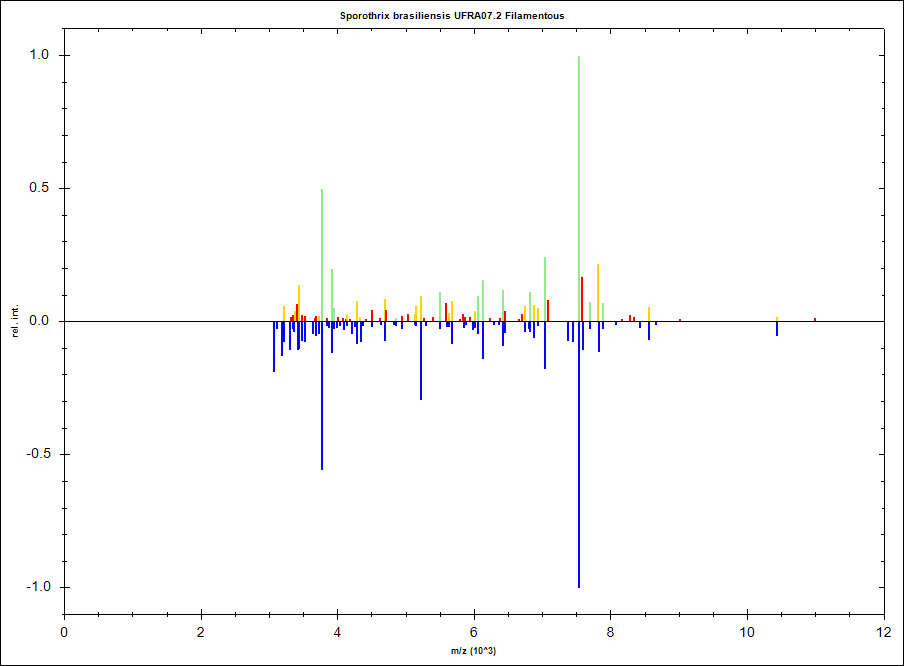


**Fig. S9** Comparison of two Main Spectra Profiles (MSPs). The upper spectrum represents UFRA07.2 yeast-like MSP and the lower, inverted spectrum represents UFRA07.2 filamentous MSP


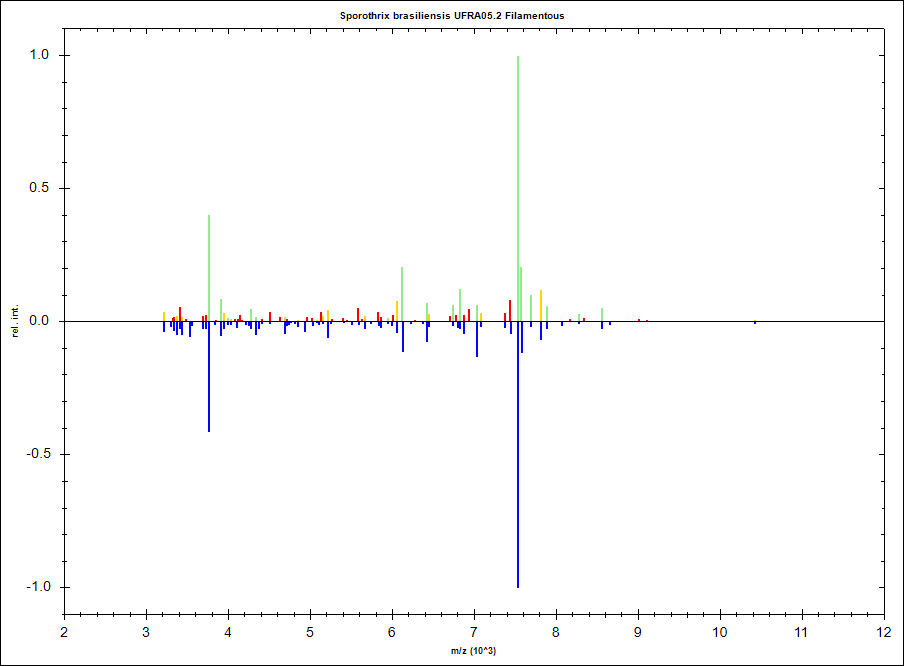


**Fig. S10** Comparison of two Main Spectra Profiles (MSPs). The upper spectrum represents UFRA05.2 yeast-like MSP and the lower, inverted spectrum represents UFRA05.2 filamentous MSP


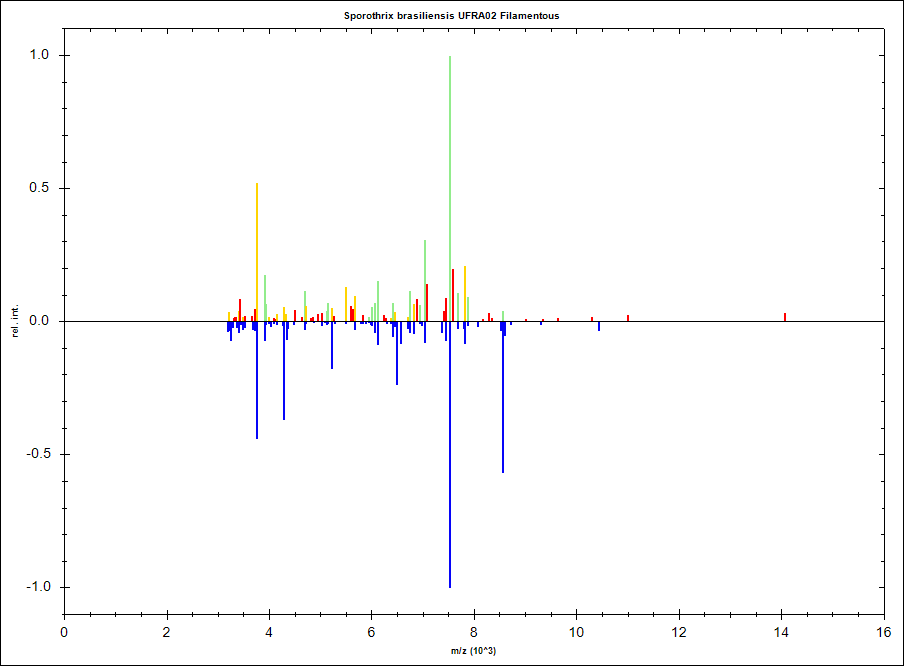


**Fig. S11** Comparison of two Main Spectra Profiles (MSPs). The upper spectrum represents UFRA02 yeast-like MSP and the lower, inverted spectrum represents UFRA02 filamentous MSP


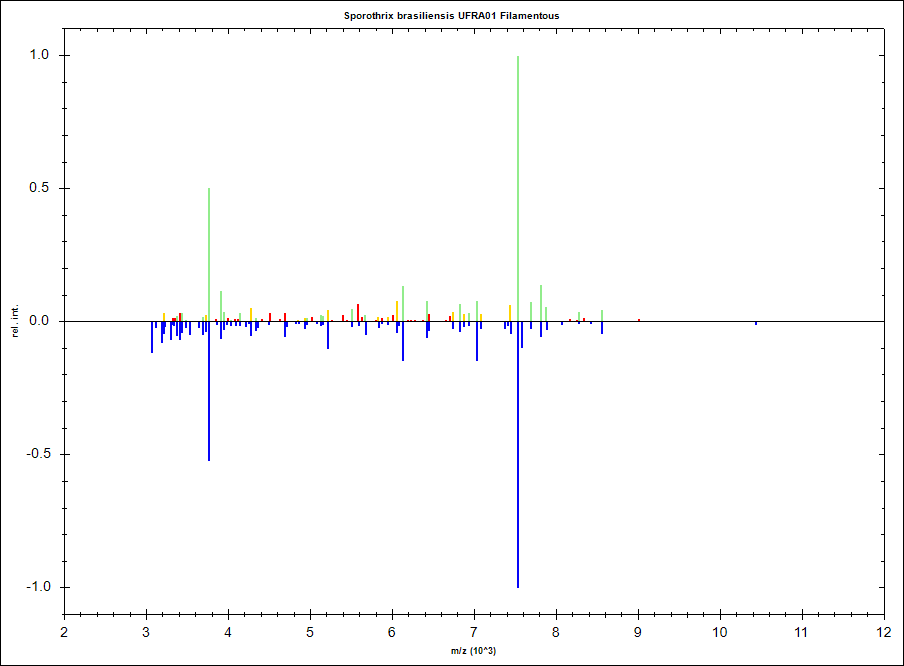


**Fig. S12** Comparison of two Main Spectra Profiles (MSPs). The upper spectrum represents UFRA01 yeast-like MSP and the lower, inverted spectrum represents UFRA01 filamentous MSP
